# Retention Challenges in Opioid Use Disorder Treatment: The Role of Comorbid Psychological Conditions

**DOI:** 10.5811/westjem50773

**Published:** 2026-01-03

**Authors:** Shu Yuan, Zi-Lin Li, Jing Hu

**Affiliations:** * Sichuan Agricultural University, College of Resources, Chengdu, China; † Medical University of the Air Force, College of Cardiovascular Medicine, Xi’an, China; ‡ Northwest University, School of Medicine, Xi’an, China

To the Editor:

We read with interest the recent paper by Dr. Seaberg and colleagues, in which they linked higher income and lower post-traumatic stress disorder scores with higher retention rates in an emergency department (ED)-based medication for opioid use disorder (MOUD) program.1 They also reported a decrease in the number of patients entering the MOUD program compared to previous years.1 We propose several suggestions to improve patient retention in MOUD programs.

Of the patients who participated in the study, 21%, 23%, 30%, and 30% indicated that they or their family members had been unable to pay for telephone service, healthcare, food, or clothing, respectively, when needed.1 Perhaps their “distrust” [with the system] may have contributed to a participant’s decision to quit the treatment program. especially in the cohort that experienced abstinence and relapse, Research conducted by Stone et al (2018) found that the overall relapse rate in patients who suffered from opioid use disorder was as high as 57%,2 while Yangchen and colleagues in 2024 found that among 1,745 patients presenting to the ED for opioid use disorder, 20% experienced a recurrent overdose.3 Difficulty during abstinence may also determine the retention rate. In those patients who experienced any recurrent overdose, the median time to first recurrent overdose was 88 days.3 If individuals with opioid use disorder encounter more difficulty in achieving successful abstinence, it may make them less likely to trust the MOUD program.

If we were to divide the participants into three groups—the people who initiated abstinence for the first time; those who experienced relapse, and those experiencing opioid overdose—we suggest that there would be large differences in their retention rates. Therefore, different treatment programs could be tailored to different groups. For example, research has shown that participants who had experienced a fentanyl-related overdose were more likely to keep naloxone nearby when using drugs compared with those who had never experienced an overdose.4 “Take-home naloxone,” thus, may not be suitable to the latter group.

Seaberg et al1 underscored that ability to regulate emotions may be a significant factor to consider in MOUD treatment and that bolstering emotion regulation skills may be an important focus of treatment. However, we believe that emotional dysregulation is not the cardinal symptom of all participants. For instance, opioid overdose may generate detrimental effects on breathing, including fatal respiratory depression.5 Personalized treatment programs such as trauma counseling and breathing improvement protocols, along with emotion regulation therapies, could be designed for each participant based on their primary symptoms. More personalized treatment measures could help to retain patients in MOUD programs.
